# Thoracoscopic closure of patent ductus arteriosus in children

**DOI:** 10.1186/1749-8090-8-S1-O244

**Published:** 2013-09-11

**Authors:** P Kołtowski, D Patkowski, C Augustyn, G Sharma, A Wesnerowicz, R CIchoń

**Affiliations:** 1 Lower Silesian Heart Centre Medinet, Wrocław, Poland; 2 Pediatric Surgery and Urology Department, Wroclaw Medical University, Wrocław, Poland

## Background

Patent ductus arteriosus is a common congenital disorder (10% of congenital disorders). Ductus Botalli closure is the realm of invasive cardiology. Inability to perform ductus Botalli closure with percutaneous methods causes, that administration of indomethacin or surgery are nowadays treatment of choice. The aim is to present an alternative in treatment with minimally invasive surgery, such as video-assisted thoracoscopic closure of PDA.

## Methods

We admitted 38 patients aged between 11 days and 7 years (65% of patients ≤ 1 year), with a weight between 700 g and 24,5 kg. During the procedure we insert trocar with optic 5mm into the pleural cavity in positioning on the right side through 4-5 intercostal space on the anterior axillary line. Secondly 3mm 2 tools are inserted through 3rd and 6th intercostal spaces on midaxillary line. We visualize posterior mediastinum and identify ductus Botalli. We close it with titanium clips in sizes depending on how large PDA is. We used 5mm clips in 27 cases (8mm,10mm in others). Average length of thoracoscopic procedure was about 40 minutes (15 minutes min,90 minutes max). After surgery we do ultrasound control.

## Results

The procedure was successful in 31 patients. Positive result of complete closure was confirmed by ultrasound diagnostic, done after the procedure. In other 6 cases, post-surgery ultrasound revealed ineffective closure and in 1 case the procedure was impossible to perform because of bad conditions of visualization. In 1 case we repeated thoracoscopic closure. In other 6 cases lateral thoracotomy was necessary. Total hospitalization time (thoracoscopic closure of patients only) varied between 2 and 5 days, while in classic surgical method it lasted on average about 7 days.

## Conclusions

Reduced invasiveness of this method seems to be more adequate than traditional lateral thoracotomy. We would like this method to be considered as an alternative treatment to the traditional lateral thoracotomy when other less invasive methods are not effective.

